# Investigating the potential of social media and citizen science data to track changes in species' distributions

**DOI:** 10.1002/ece3.10063

**Published:** 2023-05-08

**Authors:** Daisy O'Neill, Henry Häkkinen, Jessica Neumann, Len Shaffrey, Chris Cheffings, Ken Norris, Nathalie Pettorelli

**Affiliations:** ^1^ Institute of Zoology Zoological Society of London London UK; ^2^ Department of Geography and Environmental Science University of Reading Reading UK; ^3^ National Centre for Atmospheric Science University of Reading Reading UK; ^4^ Joint Nature Conservation Committee Peterborough UK; ^5^ Natural History Museum London UK

**Keywords:** biodiversity monitoring, citizen science, climate change, range shifts, species' redistribution, wildlife management

## Abstract

How to best track species as they rapidly alter their distributions in response to climate change has become a key scientific priority. Information on species distributions is derived from biological records, which tend to be primarily sourced from traditional recording schemes, but increasingly also by citizen science initiatives and social media platforms, with biological recording having become more accessible to the general public. To date, however, our understanding of the respective potential of social media and citizen science to complement the information gathered by traditional recording schemes remains limited, particularly when it comes to tracking species on the move with climate change. To address this gap, we investigated how species occurrence observations vary between different sources and to what extent traditional, citizen science, and social media records are complementary, using the Banded Demoiselle (*Calopteryx splendens*) in Britain as a case study. Banded Demoiselle occurrences were extracted from citizen science initiatives (iRecord and iNaturalist) and social media platforms (Facebook, Flickr, and Twitter), and compared with traditional records primarily sourced from the British Dragonfly Society. Our results showed that species presence maps differ between record types, with 61% of the citizen science, 58% of the traditional, and 49% of the social media observations being unique to that data type. Banded Demoiselle habitat suitability maps differed most according to traditional and social media projections, with traditional and citizen science being the most consistent. We conclude that (i) social media records provide insights into the Banded Demoiselle distribution and habitat preference that are different from, and complementary to, the insights gathered from traditional recording schemes and citizen science initiatives; (ii) predicted habitat suitability maps that ignore information from social media records can substantially underestimate (by over 3500 km^2^ in the case of the Banded Demoiselle) potential suitable habitat availability.

## Graphical abstract

To date, our understanding of the respective potential of social media and citizen science to complement the information gathered by traditional recording schemes remains limited, particularly when it comes to tracking species on the move with climate change. To address this gap, we investigated how species occurrence observations vary between different sources and to what extent traditional, citizen science, and social media records are complementary, using the Banded Demoiselle (*Calopteryx splendens*) in Britain as a case study.

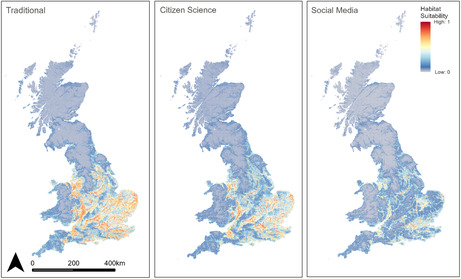

## 1 INTRODUCTION

One of the swiftest consequences of climate change is the global redistribution of species on Earth (Pecl et al., 2017; Scheffers et al., 2016). Changes in the distribution of these species on the move are anticipated to have wide‐reaching consequences for ecosystems and humans (Twiname et al., 2020; Wallingford et al., 2020). Consequently, how to best track these species as they rapidly alter their distributions has become a key scientific priority (Pecl et al., 2017). Information on species distributions is derived from biological records, which are defined as logs of species at a particular place at a certain time (Isaac & Pocock, 2015). Biological recording takes various forms and involves different contributors, methods, and information content. For a small number of taxa—namely those that are the most charismatic—structured monitoring schemes exist to provide systematic and focussed recording (Isaac et al., 2014). These include, for example for birds, the Breeding Birds Survey (Field & Gregory, 1999) and the Seabird Monitoring Programme (Walsh et al., 1995) in the UK, and the North American Breeding Bird Survey (Sauer et al., 1997). Such monitoring schemes are cost‐intensive, requiring dedicated participants, typically involve standardized protocols (Isaac et al., 2014; Pocock et al., 2015) and tend to be biased toward more developed countries (Moussy et al., 2021). Most biological recording fits within opportunistic, unstructured recording schemes. These are generally coordinated by individual specialist recording schemes or societies that collate records with a particular taxonomic focus (Pocock et al., 2015).

With technological advancements making it easier to submit records, biological recording has become more accessible to the general public (Pocock et al., 2015). Several citizen science applications, such as iNaturalist, enable individuals to submit records that can be identified through the applications' community of scientists and naturalists (Nugent, 2018). Social media moreover offer a novel source of information for answering ecological questions about biodiversity, species distributions, and the impacts of climate change. Social media websites and applications allow users to post content of any kind, offering vast amounts of untapped, freely available information when this content is relevant to the ecological questions being investigated (see e.g., Allain, 2019; Barve, 2014; Daume, 2016; ElQadi et al., 2017; Pace et al., 2019). Yet, to date, our understanding of the potential of social media to complement existing sources of biological data for monitoring species distributions and habitat suitability availability remains limited, particularly when it comes to tracking species on the move with climate change (but see Pettorelli et al., 2019). In particular, information is lacking as to how species occurrence observations differ between different sources and to what extent different types of biological records are complementary.

To address this gap, this study makes use of available species occurrence data for the Banded Demoiselle (*Calopteryx splendens*) in Britain to assess the level of complementarity and divergence between distribution and habitat suitability maps derived from traditional recording schemes, citizen science initiatives, and social media information.

The Banded Demoiselle is a highly recognizable damselfly that is currently shifting its distribution in the UK due to climate change (Brooks et al., 2007; Cham et al., 2014; Mill et al., 2010; Pettorelli et al., 2019). It is a member of Odonata (dragonflies and damselflies), and as such has a hemimetabolous life cycle consisting of egg, nymph, and adult stages (Stoks & Córdoba‐Aguilar, 2012). The nymphs are aquatic with eggs laid in aquatic plant tissue or in water, before metamorphosing into the terrestrial, flying adult stage, therefore requiring both healthy aquatic and resource‐rich terrestrial habitats (Nagy et al., 2019). It is one of a few British riverine Odonates, requiring an adequate unidirectional flow for larval respiration, therefore restricted primarily to slow‐flowing streams and rivers in lowland areas of southern Britain, although shifting further northward in recent years (Ward & Mill, 2005).

Britain makes for an excellent case study due to the vast availability of species distribution data for the UK, being arguably the most intensively recorded country on earth (Powney & Isaac, 2015), with the second greatest number of species occurrence records worldwide, behind the United States but with approximately eight times the record density (https://www.gbif.org/the‐gbif‐network, accessed April 2021). Odonata are a charismatic taxon, with a high engagement in recording both from volunteers within the UK's specialized recording scheme run by the British Dragonfly Society, as well as appealing to citizen–scientists more generally. The Banded Demoiselle, in particular, has a unique appearance and ease of species identification, being only one of two species of Demoiselle in the country with colored wings (Svensson et al., 2004), making it an ideal candidate for investigation into the use of social media and citizen science occurrence records. Based on previous work (Callaghan et al., 2018; Dickinson et al., 2010; ElQadi et al., 2017; Noviello et al., 2021), we expect (H1) habitat suitability maps derived from social media records and citizen science initiatives to significantly differ from habitat suitability maps derived from traditional records and (H2) occurrences derived from social media platforms and citizen science initiatives to be more common in urban settings compared with traditional biological recording.

## 2 METHODOLOGY

### 2.1 Species occurrence data

Species occurrence records for the Banded Demoiselle were downloaded from both the Global Biodiversity Information Facility (GBIF.org, 2021) and the National Biodiversity Network (NBN) Atlas (British Dragonfly Society Recording Scheme, 2021; National Biodiversity Network Trust, 2021). Records were selected from 2010 onwards for comparison with social media datasets. Biological records from the British Dragonfly Society (BDS) Recording Scheme (excluding records from iRecord), Local Environmental Record Centres (LERC) as well as various national and international trusts and organizations were labeled as “traditional.” Records from both the iRecord and iNaturalist platforms were labeled as “citizen science.”

Records were collected from social media platforms (Facebook, Twitter, and Flickr) using the search terms “Banded Demoiselle” and “*Calopteryx splendens*.” For Twitter and Facebook, this involved a manual search (completed between 13/01/2022 and 04/04/2022, for approximately 1.5 h a day), with biological records consisting of an identifiable photograph or video. These records included either a tagged location or a mention of location within the content of the post, as well as a date for the observation if provided (otherwise the date the content was shared). Latitude and longitude information is generally preferable, allowing for precise placement of species occurrences. However, this information was not available for Twitter or Facebook records. Around 23% of the records found included a tagged location label; however, this was typically a city or town level. As such, records from Twitter and Facebook were manually checked and georeferenced by determining all the 1‐km British National Grid squares that covered the spatial extent of the location description provided by the user. Although more imprecise than tagged geolocations, this ensured that the location information included was where the observation occurred (as opposed to where the photograph was uploaded). Searches yielded 95 results from Twitter and 331 from Facebook, which covered 295 and 867 1‐km grid squares, respectively. These 1‐km grid squares were included as Banded Demoiselle occurrences in subsequent species distribution models (SDMs). For each social media occurrence, spatial precision (estimated to the nearest km^2^) was recorded in the final dataset. For Flickr, records were collated with the Flickr application programming interface (API) using the Flickr.photos.search (http://www.flickr.com/services/api/flickr.photos.search.html). Initial searches yielded 1316 results with location information as well as date recorded and posted that were extracted in R using the package FlickrAPI (Ando & Pousson, 2022). These results were then manually verified, with 1223 observations remaining once records observed outside the relevant time frame or study location as well as irrelevant or misidentified observations were removed. For each data type, occurrence records were cleaned using the R package CoordinateCleaner to flag and remove erroneous or duplicate results (Zizka et al., 2021). Potential data entry errors and failed georeferencing were flagged by checking for equal latitude and longitude values as well as zeros in the coordinates; coordinates matching country centroids and biodiversity institutions were also removed to ensure occurrences with imprecise georeferencing or captured individuals were excluded (Zizka et al., 2019).

The low precision of Facebook and Twitter social media data is a potential source of error during modeling as it may overestimate the current range and therefore the range of suitable habitats. The location descriptions provided varied in precision; some observations detailed exact locations that could be prescribed to individual 1‐km grid squares, whereas others described wider locations covering several km grids. As such, we performed additional sensitivity analyses using several alternative subsets of the social media data; in these, the dataset was filtered to only include points with a spatial precision of at least 1, 2, 5 and 10 km^2^, respectively. Results of these models were compared with those that used all social media data points, using Spearman's correlation to check for sensitivity of results to differing thresholds of spatial precision, as well as spatial assessment of uncertainty between different cropped datasets.

### 2.2 Environmental data

The set of environmental variables considered to shape the distribution of Banded Demoiselle in the UK included climatic conditions, topography, landcover type, vegetation productivity, and level of urbanization. Monthly minimum and maximum temperature as well as monthly precipitation for the period 1990 to 2020 were accessed from the Met Office at a 1‐km resolution (Met Office et al., 2022) and used to generate a series of monthly average bioclimate variables using the biovars function in the R package dismo (Hijmans et al., 2021), under the assumption that species' ranges respond to the long‐term averages of climate conditions (Taheri et al., 2020). These climate variables represent annual trends, seasonality, and limiting environmental factors and as such are designed to be biologically meaningful, being widely used for SDMs (Manzoor et al., 2018), and informative for Odonatan distributions (Abbott et al., 2022; Collins et al., 2017).

Slope was extracted from the Ordnance Survey (OS) Terrain 50 Digital Terrain Model (DTM) accessed from EDINA Digimap (OS Terrain 50, 2013); slope is important for Odonata species due to its influence on water velocity, O_2_ content, weathering, channel substrate size, and organic matter composition (Collins & McIntyre, 2015) and of particular importance to the Banded Demoiselle that favors slow‐flowing rivers.

To capture the aquatic element of the Banded Demoiselle's niche, the percentage cover at 1‐km resolution of the freshwater aggregate class was extracted from the Centre for Ecology and Hydrology (CEH) 2015 Land Cover Map accessed from EDINA Digimap (Land Cover Map 2015, 2017). A Water and Wetness Probability Index (WWPI) product coordinated by European Environment Agency (EEA) Copernicus program was also acquired which indicates the occurrence of water and wet areas as a continuous probability at 20‐m resolution based on observations between 2009 and 2015 (Langanke et al., 2018).

Normalized Difference Vegetation Index (NDVI; Pettorelli, 2013) Long Term Statistics (LTS) version 2.2. were also included from the Copernicus Global Land Service (CGLS) at a 1‐km resolution (Toté et al., 2021). These statistics include the minimum, median, maximum, average, and standard deviation calculated from 10‐daily NDVI values throughout 1999 to 2017 derived from Spot‐4, Spot‐5, and Proba‐V satellite imagery. The NDVI gives an indication of “greenness” and therefore is likely to be influential in odonatan distribution. In addition, the CGLS 100‐m resolution tree cover density for the reference year 2012 was included (European Environment Agency, 2018). These should account for the influence of vegetation on the Banded Demoiselle distribution, where vegetation influences territory selection and where eggs are laid into aquatic emergent vegetation (Ward & Mill, 2005). To account for varying levels of urbanization, annual composites of visible night light version 2 were acquired for the years 2014 to 2018 from the Earth Observation Group (Elvidge et al., 2021) and averaged across these years.

Predictor variables were reprojected to the British National Grid and aggregated to a 1‐km resolution where needed using the functions projectRaster and aggregate in R package raster (Hijmans & van Etten, 2012). All predictors were scaled to a mean of zero and a standard deviation of one. Predictor distributions were checked for any significant skew and a log transformation applied where a strong skew was identified.

The Pearson's correlation coefficient was calculated between each pair of predictor variables and where the coefficient was greater than 0.7, only one variable was retained. Including covarying predictors above this threshold results in increased uncertainty and disagreement among projections (Brun et al., 2019; Dormann et al., 2012). In cases where it was unclear which covarying predictor should be kept, two separate models were run with each set of covarying predictors, and the variable that contributed to more accurate model fit (assessed by true skill statistic [TSS] and the area under the receiver operating characteristic curve [AUC]) was kept. As a final check to ensure no correlated predictor variables were included, the Variable Inflation Factor (VIF), a measure of multicollinearity, was calculated for each occurrence dataset before model computation, to ensure that VIF was less than six, which is deemed acceptable (Guisan et al., 2017).

A preliminary set of SDMs was implemented through biomod2 with a dataset of all species occurrence records and all environmental variables to examine variable importance and guide predictor selection. Importance was determined by computing the Pearson's correlation between predictions made with a given variable and with the variable replaced with a randomized input, with variable importance averaged from five permutations. These preliminary screening steps resulted in a final set of predictors consisting of mean annual temperature, isothermality, mean temperature of the wettest and driest quarters, total annual precipitation, slope, percentage freshwater cover, WWPI, mean NDVI, and percentage tree cover.

### 2.3 Sampling effort

Species distribution models rely on the assumption that sampling effort and probability of detection are approximately even over a given area. However, this is often not the case, especially for opportunistically sampled data such as in citizen science projects and social media, and as such sampling bias can severely distort results (Bird et al., 2014; Johnston et al., 2021). A typical way to counteract this is with a target‐group background approach (Phillips et al., 2009), which uses sampling from other related taxonomic groups to give a broad overview of sampling effort over an area. In this study, this approach was not possible as acquiring an equivalent sampling background for social media data is extremely difficult, if not impossible, due to the time and computational workload involved. Instead, we used a “bias covariate correction” method (Chauvier et al., 2021; Warton et al., 2013), where several proxies for sampling effort are used to correct for areas of bias. We therefore included several sampling effort predictors in our models, namely distance to major population center, distance to nearest road, and population density. Shapefiles for major population centere were downloaded from the Office for National Statistics (2021) and the Scottish Government SpatialData.gov.scot (2022), and the distance from each 1‐km grid cell in our study area to the nearest city was calculated. Spatial line data for roads were based on OpenStreetMap Data Extracts, as processed by Geofabrik GmbH (2023), using the latest road data available for the UK as of February 13, 2023; for each grid cell in the study area, we calculated how far they lay from the nearest road. Residential population density was downloaded from the Environmental Information Data Centre (2023) at 1‐km resolution. Predictor covariation was assessed, and a preliminary set of models was run to check for variable importance (following same methods as for environmental variables). Where sampling effort variables were important (1 − *r* > 0.05, where r is the Pearson's correlation coefficient), they were retained in the final model. When final projections were made, these variables were set to the median value for a given layer across the study area, to compensate for the potential effect of sampling effort following the protocol of Warton et al. (2013).

### 2.4 Species distribution modeling

Ensemble SDMs for the Banded Demoiselle were implemented using the R biomod2 package (Thuiller et al., 2021) for each species occurrence dataset. There was no a priori reason to select one family of models over another, so all were trialed and compared in terms of habitat suitability outputs, performance metrics provided by biomod2 (accuracy, bias, TSS, and AUC), and variance in estimated response curves. Since all performed similarly and showed broadly similar outputs (Figure S3), ensemble model results were built with output from all high‐performing models, regardless of family. As such, a set of six modeling techniques were selected including three machine‐learning methods, generalized boosting model, random forest, and maximum entropy; two regression methods, generalized linear model, and multiple adaptive regression splines; and finally, a recursive partitioning method, classification tree analysis. For all modeling algorithms, the default biomod2 settings were used.

For each dataset (traditional, citizen science, and social media), 10,000 pseudo‐absence points were randomly selected from the background data, a quantity approximately matching the most numerous occurrence dataset, to be broadly appropriate across SDM techniques (Barbet‐Massin et al., 2012). To ensure pseudo‐absence composition was not impacting results, preliminary SDMs were computed with 5, 10 and 15 sets of pseudo‐absence points. Agreement was high overall across all statistical metrics used (Table 2) and did not differ significantly between runs with different numbers of pseudo‐absence sets. As each dataset was large and computationally taxing, all final models were run with five pseudo‐absence datasets. To reduce the potential of selecting pseudo‐absences within the same niche as presences, pseudo‐absences were placed at least 1.5 km away from any observed occurrences that have a coordinate uncertainty of up to 1 km.

Several validation models were created, where 20% of the species occurrences, including both presence and pseudo‐absence points, were set aside for evaluation. Model performance was assessed with TSS, which provides a threshold‐independent measure of accuracy (Allouche et al., 2006). TSS has several documented drawbacks (Leroy et al., 2018), notably its dependence on prevalence; however, we chose to use a balanced approach where the number of pseudo‐absences was set to match the number of presences, as this reduces the chance of bias when using TSS results, allows easier comparison between different models as prevalence is held constant, and is the recommended approach when attempting to maximize discrimination in SDMs (Steen et al., 2020). Several alternative metrics were also calculated to provide an overall summary of performance and potential bias. These included AUC; frequency bias, the ratio between observed and predicted presences; accuracy, the fraction of occurrences correct; and finally Cohen's Kappa coefficient, a measure of model accuracy which corrects for accuracy expected to occur by chance (Allouche et al., 2006). This process was repeated five times, splitting the occurrences into five random training and testing sets of 80% and 20%, respectively, balancing the ratio of presence and pseudo‐absence points, to ensure that their composition was not having any impact on model accuracy. Ensemble models were built combining all individual models with a TSS value greater than 0.6, considered to be useful to excellent (Komac et al., 2016), and weighing model contribution according to their TSS.

The evaluation results are based on the internally validated models, whereas the final projections presented throughout the manuscript are based upon all available occurrence data, without any presences or pseudo‐absences set aside for internal validation. This is to ensure the final parameter estimates are built with the maximum information and therefore lower uncertainty in parameter estimates and projections. As our validation models were robust, we verified that the final full models were sufficiently similar to the validation models so as to ensure the final full models were similarly robust. We verified this using a Spearman's correlation between the projected habitat suitability of five validation models and the final models for each data source.

Each ensemble model of habitat suitability was converted into binary presence–absence maps; thresholds were selected to maximize the combined sensitivity and specificity scores (Liu et al., 2016). Pair‐wise comparisons were carried out to compare predictions between models based on different occurrence datasets, computed for both habitat suitability predictions and binary presence–absence maps. Similarity between predictions was calculated using Spearman's correlation tests.

Banded Demoiselle habitat was further analyzed by extracting the proportion of predicted presences within each of the 10 aggregate classes of the CEH 2015 land cover map accessed from EDINA Digimap (Land Cover Map 2015, 2017). This included a built‐up areas and gardens class, to compare suitable habitat within urban areas across occurrence data types.

## 3 RESULTS

**FIGURE 1:**
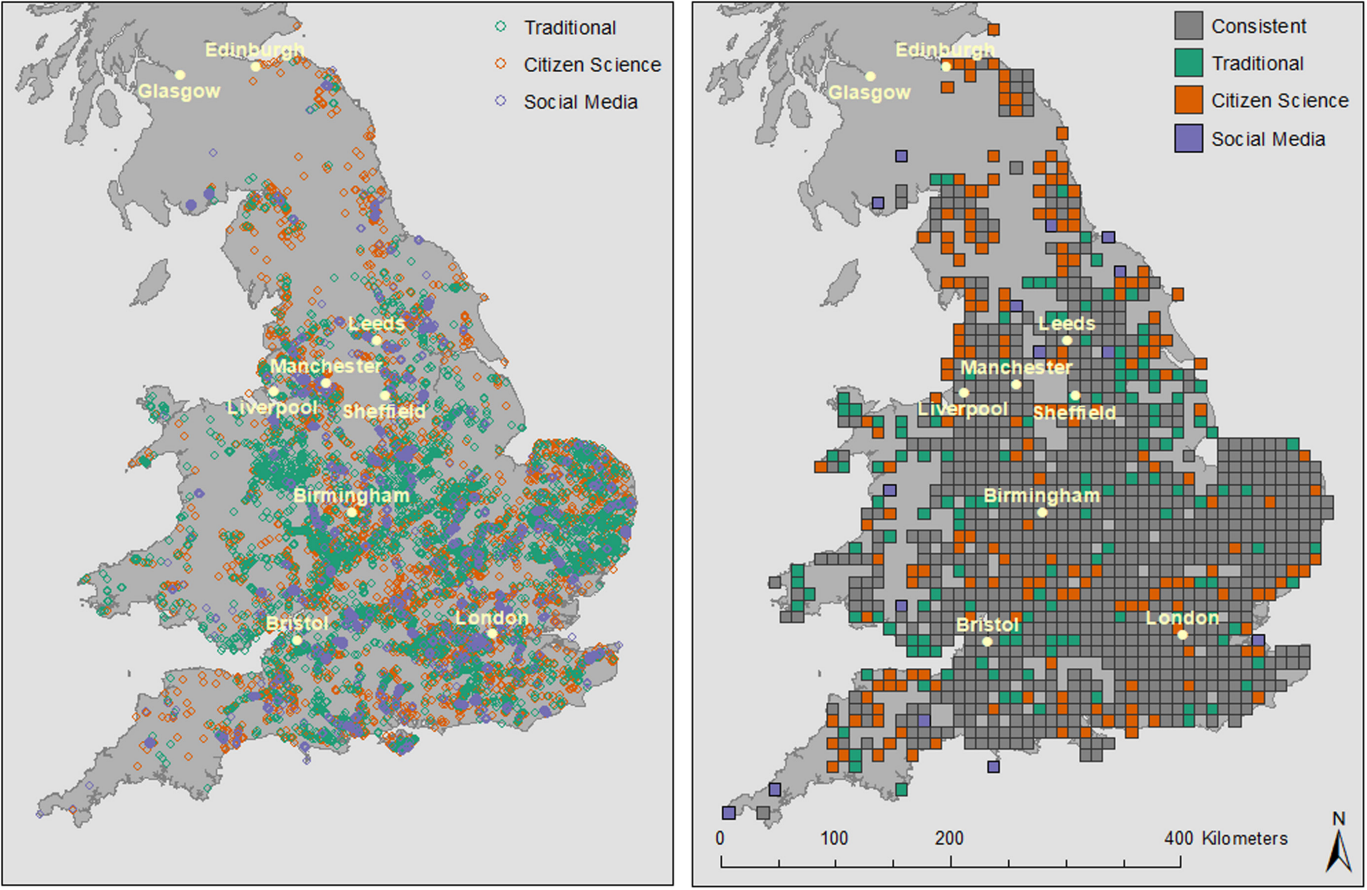
Distribution of traditional, citizen science, and social media species occurrence records (left) and consistencies and differences when gridded to the 10 km British National Grid (right). Population centers with more than 500,000 people have been highlighted.

A total of 17,831 observations of the Banded Demoiselle were collected (Table 1). When gridded to the 1 km^2^ British National Grid, at the same resolution as the predictor variables, a large proportion of the total number of grid cells where presence was reported for each occurrence type, were unique to that data type; ~61%, ~58% and ~49% for citizen science, traditional and social media, respectively. When aggregated to 10 km^2^, the difference becomes less stark (Table 1; Figure 1).

**TABLE 1:** Total number of occurrence records collected for each type, including the number of 1 and 10‐km British National Grid squares. For each type, the number and proportion of grid squares where observations were unique to that type is given.

Type	Details	Total records	1 km grids	Unique 1 km grids	10 km grids	Unique 10 km grids
Traditional	BDS; LERCs; National Trusts/Organizations	6749	4211	2424 (57.6%)	908	184 (20.3%)
Citizen science	iRecord; iNaturalist	9646	5075	3100 (61.1%)	982	136 (13.8%)
Social media	Facebook; Flickr; Twitter	2026	1480	726 (49.1%)	421	15 (3.6%)

Abbreviations: BDS, British Dragonfly Society; LERC, Local Environmental Records Centre.

The TSS and Kappa scores across all SDMs were greater than 0.6, while all AUC values exceeded 0.85, indicating good model performance (Table 2). Model performance was broadly similar across all data sources (Table 2). Accuracy and bias values were similar across data types, and high across all models. Validation models were representative of the final models as Spearman's correlation coefficients between validation and final models were greater than 0.98 in all cases.

**TABLE 2:** Evaluation statistics for the ensemble models averaged from validation runs for each species occurrence data type, including the true skill statistic (TSS), the area under the receiver operating characteristic curve (AUC), Cohen's *κ* coefficient, sensitivity, and specificity. Values in brackets are the standard deviation across the five validation runs.

Occurrence dataset	TSS	AUC	*κ*	Accuracy	Bias
Traditional	0.60 (0.05)	0.88 (0.03)	0.60 (0.05)	0.80 (0.02)	0.99 (<0.01)
Citizen science	0.66 (0.05)	0.91 (0.02)	0.65 (0.04)	0.84 (0.02)	0.99 (<0.01)
Social media	0.66 (0.04)	0.90 (0.02)	0.62 (0.05)	0.86 (0.02)	0.99 (0.02)
All	0.65 (0.05)	0.90 (0.02)	0.61 (0.05)	0.87 (0.02)	1.00 (<0.01)

Annual mean temperature and percentage freshwater cover were highly ranked variables for all three data sources (Table S1) and were found to be important in all three models (1 − *r* > 0.1, where r is the Pearson's correlation coefficient). In addition, summed annual precipitation was found to be highly important in citizen science and traditional SDMs, but not for social media. Distance to the nearest roads was an important predictor for social media SDMs but was less important when using traditional or citizen science data sets. For full details on variable importance for all three data sources, see Supporting Information (Table S1). The breadth of suitable environmental conditions and response curves were broadly similar across data types (Figure S1).

Distance to roads was the only covariate of sampling effort that was found to have any effect on the models, and outputs shown here are made following correction for sampling effort. Comparisons with uncorrected models are included in Supporting Information (Figure S2), and significant differences in suitability for social media SDMs can be seen around major population centers including London, Manchester, and Birmingham.

Social media had higher spatial uncertainty than data from other sources, so several sensitivity tests were carried out. SDMs were constructed with points with a spatial precision of at least 1, 2, 5, 10 km^2^, respectively, and compared to models constructed with the full data set. The most dissimilar models were those built with all data and those built with 2 and 1 km^2^ precision data (Spearman's coefficient: 0.96 and 0.97 respectively; Table S2). All models were broadly similar (Figure S3), though uncertainty was higher around major population centers and coastal areas. The results presented here are for models built with all data.

**FIGURE 2:**
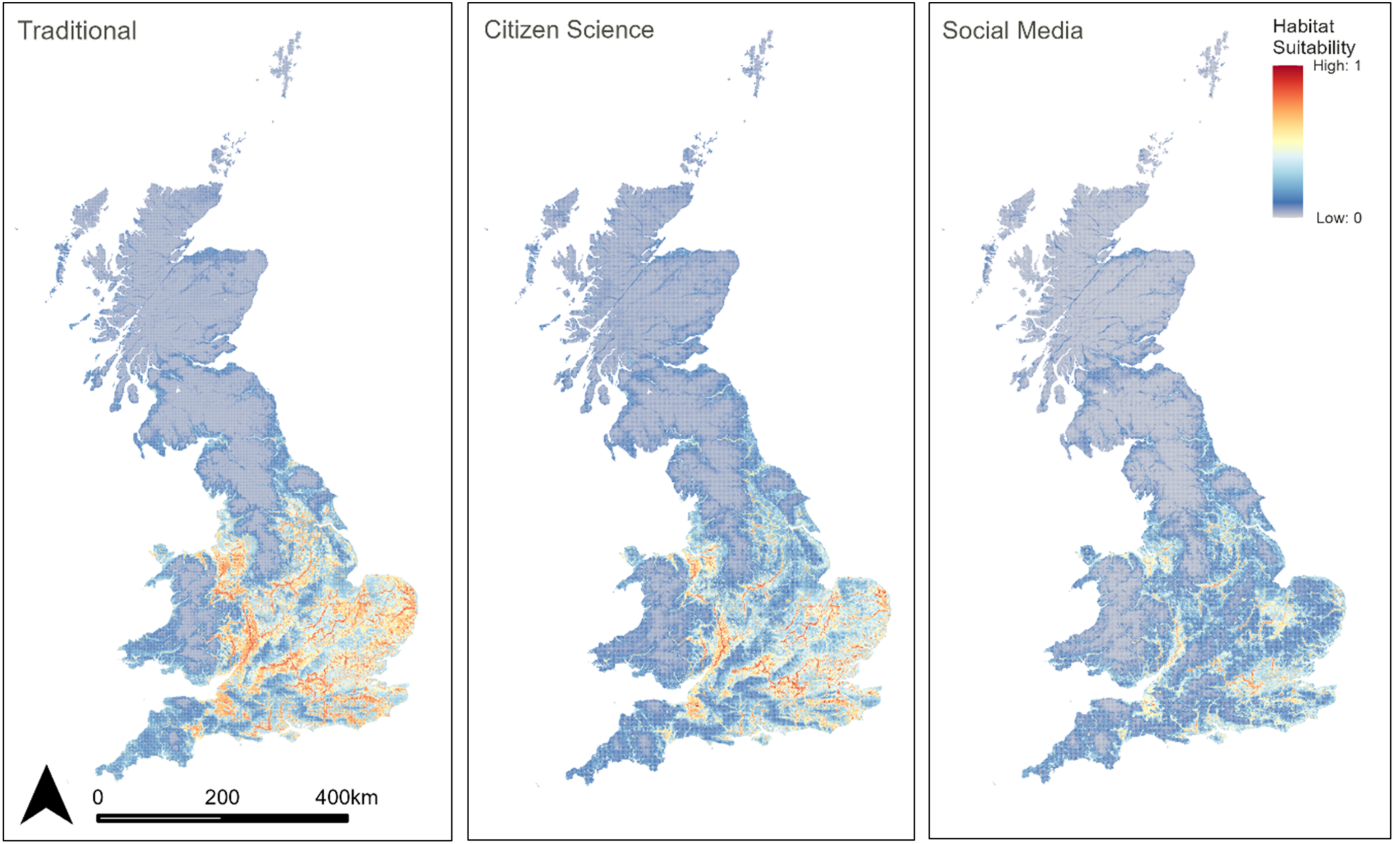
Projected habitat suitability index according to weighted mean ensemble models computed based on traditional (left), citizen science (middle) and social media (right) observations.

Under our ensemble model based on traditional occurrence records, around 50,800 km^2^ (21.71%) of Great Britain's landmass is predicted suitable for the Banded Demoiselle; this is compared to ~54,600 km^2^ (23.33%) based on citizen science records and ~41,500 km^2^ (17.73%) based on social media records (Figure 2). As expected, using all collected data led to the greatest total projected area of suitable habitats for the Banded Demoiselle (~57,600 km^2^, 24.60%). Suitable habitats for the Banded Demoiselle were predicted to primarily include arable lands (37.9% to 48.5% of total suitable area), improved grasslands (32.6% to 33.5%) and built‐up areas (11.8% to 21.0%), with only a small proportion of suitable areas found within broadleaf woodlands (3.1% to 3.8%). The study area was similarly dominated by the arable and improved grasslands land cover types, covering together 57.6% of the total area (Table 3).

**TABLE 3:** Coverage of land cover classes for the Great Britain study area and the predicted suitable habitat for the Banded Demoiselle according to ensemble species distribution models based on different types of species occurrence records.

Class	Study area	Traditional	Citizen science	Social media
**Improved grassland**	**31.2% (73,084)**	**33.5% (17,003)**	**32.6% (17,770)**	**33.4% (13,854)**
**Arable**	**26.4% (61,865)**	**48.5% (24,636)**	**47% (25,642)**	**37.9% (15,747)**
Mountain, heath, bog	15.4% (35,926)	0.4% (195)	0.4% (244)	0.4% (181)
Semi‐natural grassland	9.5% (22,113)	0.7% (339)	0.6% (351)	0.8% (334)
**Built‐up areas and gardens**	**6.6% (15,394)**	**11.8% (6004)**	**13.7% (7455)**	**21% (8716)**
Coniferous woodland	6.1% (14,303)	1.0% (502)	1.1% (574)	1.0% (400)
**Broadleaf woodland**	**2.5% (5919)**	**3.1% (1552)**	**3.4% (1850)**	**3.8% (1571)**
Coastal	1.2% (2831)	0.5% (230)	0.5% (290)	0.7% (284)
Freshwater	0.6% (1512)	0.6% (321)	0.7% (372)	0.9% (372)
Saltwater	0.4% (1042)	0.0% (24)	0.1% (36)	0.1% (36)

*Note*: Percentages are given of total study area and total predicted suitable habitat, with values in brackets being the total area in kilometers squared. Bold text is used to indicate land classes where Banded Demoiselle suitable habitat dominates (where total suitable area > 1000 km^2^).

**FIGURE 3:**
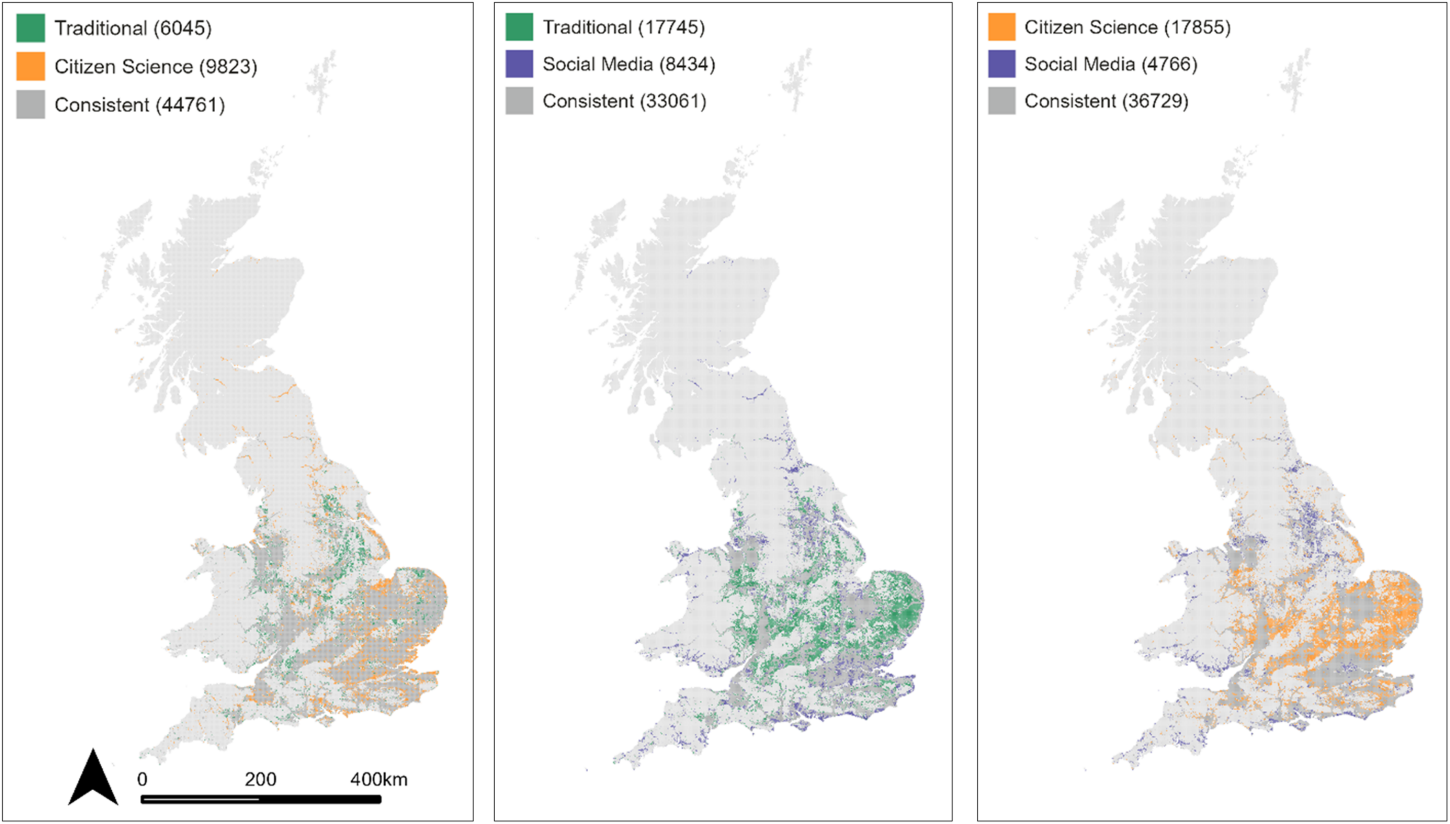
Pairwise comparison between projected suitable habitat for the Banded Demoiselle according to different data types. Predictions were converted to binary presence–absence maps using the threshold that maximized the true skill statistic for each ensemble model. Values in brackets indicate the total consistencies and differences between predicted suitable habitats in terms of the number of 1‐km pixels and therefore total area in km^2^.

Spearman's correlation coefficients between habitat suitability maps based on different record types were greater than 0.85 for all pairs of occurrence datasets. Projections based on traditional and citizen science records were the most correlated (0.95) while projections based on traditional and social media records were the least correlated (0.87, Table 4). The area consistently expected to be suitable for the Banded Demoiselle was estimated to cover 44,761 km^2^ when comparing models based on traditional and citizen science records; but this area was expected to only cover 33,061 km^2^ when comparing models based on traditional and social media records. In the latter situation, 17,745 km^2^ of suitable habitats was uniquely identified by traditional records while 8434 km^2^ of suitable habitats was uniquely identified by social media records. The area uniquely identified as suitable by traditional records primarily covers the southern lowlands, while the area uniquely identified as suitable by social media records covers the southwest, south Wales, coastal areas around the south of the UK, the northeast and Scotland (Figure 3). A greater proportion of projected suitable habitat was found within built‐up and urban areas when considering social media records (21%) than citizen science (13.7%) and traditional data (11.8%).

**TABLE 4:** Spearman's correlation between models derived from different species occurrence records. Above diagonal values are the correlation between binary presence–absence maps and below diagonal the correlation between habitat suitability projections.

Habitat suitability maps	Binary (presence/absence) maps		
	Traditional	Citizen science	Social media
**Traditional**	1	0.805	0.651
**Citizen science**	0.952	1	0.714
**Social media**	0.870	0.928	1

## 4 DISCUSSION

This study offers a unique assessment of the level of complementarity and divergence between habitat suitability distributions derived from traditional recording schemes, citizen science initiatives, and social media information. Our results show that (i) social media records provide insights into the Banded Demoiselle distribution and habitat preference that are different from, and complementary to, the insights gathered from traditional recording schemes and citizen science initiatives; (ii) predicted habitat suitability maps that ignore information from social media records substantially underestimate (by over 3500 km^2^) potential suitable habitat availability.

The use of social media to extract species occurrence observations and inform ecological research and wildlife management is a relatively new concept, with a few cases where such methods have been investigated both for native (Barve, 2014; ElQadi et al., 2017; Pace et al., 2019) and non‐native species (Allain, 2019; Daume, 2016). Social media data can greatly extend the number of occurrence records available to ecologists (Allain, 2019) and, in the case of countries with limited resources, provide an alternative to costly specialized recording schemes and citizen science campaigns (Di Minin et al., 2015). Our study demonstrates that there is much value in capitalizing on this new type of information: Even though substantially less numerous than the other data types overall, 49% of the Banded Demoiselle presences derived from social media platforms were unique to social media, enabling us to capture a broader perspective on the species' habitat preferences. Our conclusions resonate with previous research by ElQadi et al. (2017) who found that Flickr observations of honeybees in Australia (i) extended the known distribution based on traditional records towards urban centers, and (ii) represented tourist areas in remote locations that were not depicted by traditional records (ElQadi et al., 2017). Together, these results suggest that spatial patterns in social media recorder activity tend to be different from the patterns found among recorders involved with traditional and citizen science data collection.

Our findings demonstrate that social media projections of Banded Demoiselle habitat cover a larger proportion of built‐up areas and gardens than traditional recording. This may potentially be an artifact of sampling bias, but it may also indicate that these urban areas provide important habitats for Banded Demoiselles, something that could be underestimated without the consideration of social media observations. The proportions of the other land cover types were largely consistent between data types, with predicted Banded Demoiselle habitat dominated by arable and improved grasslands. This contradicts previous findings that agriculture, managed land, and excessive grazing do not provide suitable Banded Demoiselle habitat due to diminished bankside vegetation (Lowdon, 2015; Ward & Mill, 2005). The coarse spatial resolution considered in this study, together with the fact that our study area is heavily dominated by these landcover types (covering 57.6% of our study area), may explain such results.

Sourcing information on species presence from social media platforms is not straightforward, and the amount of information garnered can be quite limited. For example, the manual Facebook and Twitter searches yielded 331 and 95 results, respectively, for Banded Demoiselle. These numbers are comparable with similar studies that have extracted species occurrence records from Facebook, such as the ones by (i) Campbell and Engelbrecht (2018) that gathered 1239 observations for 34 species of baboon spiders across Southern Africa (around 36 records per species), (ii) Rocha et al. (2017) that sourced 369 records of the Eurasian red squirrel in Portugal, and (iii) Havlin et al. (2017) that collected 30 observations of red‐necked wallabies on the Isle of Man, UK. These investigations all used specific Facebook pages set up by the scientists and dedicated to encouraging submission of records for their target species. In our case, biological records were gathered from existing platforms, which may partially explain the low numbers of records we were able to source. Although requiring greater effort and longer term management, dedicated pages may yield a greater number of results as well as being a more active way of engaging communities with biological recording.

Acquiring biological records from Flickr was aided by the use of an API that allows for an automated search of visual content and extraction of information on associated location and date. Using this API for the Banded Demoiselle yielded 1316 initial results instantly, providing both a faster method to access information in comparison to other social media platforms investigated as well as yielding a greater total number of observations. Although the initial search was rapid in comparison with manual searches on Twitter and Facebook, the subsequent manual verification of the data was, however, time‐consuming. The R package CoordinateCleaner (Zizka et al., 2021) provided a means to rapidly flag and remove likely erroneous records, such as those assigned to country centres and biodiversity facilities, as well as identify outliers and duplicate observations. The difficulty with Flickr API searches is that this can yield observations where species are incorrectly identified, alongside content where the species name is mentioned in another context without any intention to indicate presence of the species. This verification step was proven to be important in our case, leading to the removal of 92 sightings (~7% initial results) despite the deliberate selection of an easily identifiable species. For other species, results may be even less reliable, such as for two bumblebee species in Australia where only 65% and 68% of the occurrences extracted from Flickr by ElQadi et al. (2017) were correctly identified. Research to identify alternatives to manual verification process is needed (ElQadi et al., 2017).

Citizen science has become an invaluable and cost‐effective source of species occurrence records (Noviello et al., 2021). Nevertheless, a number of concerns remain about the accuracy and quality of citizen science data due to variability in volunteers' level of experience and expertise (Aceves‐Bueno et al., 2017), with previous studies finding a lower performance of SDMs based on citizen science data compared with systematic surveys (Tiago et al., 2017) and suggesting filtering citizen science data according to data quality and information content for more accurate SDMs (Van Eupen et al., 2021). In our case, however, all SDMs performed adequately, and habitat suitability maps derived from traditional and citizen science sources were the most congruent. These comparable results from citizen science and traditional observations are likely partially a result of improved data validation within citizen science initiatives (Dickinson et al., 2010), with, for example, iNaturalist crowdsourcing verification from users within the platform and iRecord verification largely being performed by volunteers associated with national recording schemes, such as within the BDS—likely the same county recorders that oversee and verify the BDS's own records. Moreover, both the BDS and citizen science records are largely collected with an unstructured and opportunistic framework.

A number of limitations to our study should, however, be acknowledged. First, this work was performed at a relatively coarse resolution; fine‐scale and more sophisticated hydrological and hydraulic predictor variables could prove advantageous for Odonatan modeling (Collins & McIntyre, 2015). Second, modeling approaches focused on rivers and water bodies, as opposed to approaches based on gridded variables as well as the combination of stream‐only and terrestrial‐only model processes, have been previously encouraged when aiming at identifying suitable habitats for freshwater species such as Banded Demoiselle (Collins & McIntyre, 2015). However, such an approach was not feasible here, particularly as the vast majority of occurrences collated were for the terrestrial adults as opposed to aquatic nymphs. Third, biotic variables have been increasingly employed to improve predictive ability of SDMs (Yates et al., 2018), with competition and intraguild predation particularly significant constraints on Odoanata distributions (Pélissié et al., 2022); however, inclusion of these interactions as predictors for Banded Demoiselle habitat was beyond the scope of this study due to the quantity of interactions possible. As such, these biotic factors are likely to modify the projected potential suitable habitat throughout Britain in practice. Fourth, most of the Twitter occurrences lacked geo‐location information and so, along with Facebook, relied on location information within the content that lacked precision compared with traditional occurrences. In this study, there was little evidence that using lower precision data significantly affected results, verified through several sensitivity analyses, but this is unlikely to be universally true and should be treated carefully. Fifth, for social media, when the location of the observation was not explicitly detailed an assumption was made that the tagged location provided information as to where the picture was taken; this cannot be confirmed and therefore adds a level of uncertainty regarding the reliability of social media data. Sixth, it is possible that individuals could report Banded Demoiselle occurrences with multiple sources, leading to duplicates that may affect the correlation and similarities between data types. Seventh, we found evidence that sampling bias can be more prevalent in citizen science and social media data, than in more traditional sampling surveys. There are numerous published methods of compensating for these issues (Chauvier et al., 2021; Ranc et al., 2016; Stolar & Nielsen, 2014), some of which were used here, but established methods may be difficult to carry out for limited social media data. Finally, while providing a compelling case for employing social media data for the Banded Demoiselle, the generality of our conclusions requires further investigation to determine whether our findings apply for other species, particularly those that are perhaps more difficult to identify by nonexperts.

## 5 CONCLUSION

Public participation has become commonplace within scientific research aimed at biodiversity monitoring and conservation, enabling access to a monumental breadth of data on species occurrence unobtainable otherwise. Our study offers a compelling illustration of the value of alternative sources of traditional biological records and highlights, in particular, the value of ecological information derived from social media data as an inexpensive and complementary source of species occurrence data. This source of freely available information can be exploited to capture a more complete understanding of species habitat preferences, appreciate the influence of urban settings, and gain insights that cannot be attained from traditional recording alone. We believe further development of APIs to gather social media information, technologies for automated verification, and greater adoption of available geo‐tagging facilities, would further broaden the scientific application of social media.

## AUTHOR CONTRIBUTIONS

**Daisy O'Neill:** Conceptualization (lead); data curation (lead); formal analysis (equal); investigation (equal); methodology (equal); validation (equal); visualization (equal); writing – original draft (lead). **Henry Häkkinen:** Formal analysis (equal); investigation (equal); methodology (equal); validation (equal); visualization (equal); writing – review and editing (equal). **Jessica Neumann:** Supervision (lead); writing – review and editing (equal). **Len Shaffrey:** Supervision (supporting); writing – review and editing (equal). **Chris Cheffings:** Supervision (supporting); writing – review and editing (equal). **Ken Norris:** Supervision (supporting); writing – review and editing (equal). **Nathalie Pettorelli:** Conceptualization (lead); funding acquisition (lead); supervision (lead); writing – original draft (lead).

## CONFLICT OF INTEREST STATEMENT

None declared.

## Supporting information


Appendix S1.

## Data Availability

The social media data that support the findings of this study are openly available on Dryad, https://doi.org/10.5061/dryad.0gb5mkm61.
