# Supplementary material for: Investigating the potential of social media and citizen science data to track changes in species' distributions
Source: Ecol Evol. 2023 May 8;13(5):e10063. doi: 10.1002/ece3.10063 (PMC10166650; doi:10.1002/ece3.10063)
Supplement: Supplementary file 1 — Appendix S1. [file ECE3-13-e10063-s001.docx]

**Supplementary Information**

Table S1. Variable importance for all variables included in final SDM models. Figures show median values across all runs, figures in brackets are the standard deviation of all estimates.

| Variable | Traditional | Citizen Science | Social Media |
| --- | --- | --- | --- |
| Annual mean temperature | 0.367 (0.094) | 0.243 (0.083) | 0.206 (0.086) |
| Annual precipitation | 0.132 (0.042) | 0.162 (0.039) | 0.088 (0.045) |
| Percentage freshwater cover | 0.119 (0.058) | 0.137 (0.069) | 0.125 (0.066) |
| Distance to nearest road | 0.064 (0.025) | 0.087 (0.036) | 0.135 (0.058) |
| Water and wetness probability index | 0.059 (0.031) | 0.05 (0.019) | 0.082 (0.037) |
| NDVI | 0.044 (0.022) | 0.041 (0.026) | 0.039 (0.025) |
| Isothermality | 0.029 (0.016) | 0.012 (0.01) | 0.028 (0.026) |
| Mean temperature of driest quarter | 0.015 (0.011) | 0.007 (0.01) | 0.009 (0.012) |
| Mean temperature of wettest quarter | 0.012 (0.015) | 0.017 (0.012) | 0.008 (0.01) |
| Distance to nearest city | 0.009 (0.01) | 0.009 (0.008) | 0.014 (0.023) |
| Mean night light | 0.006 (0.008) | 0.019 (0.017) | 0.075 (0.028) |
| Slope | 0.006 (0.008) | 0.003 (0.004) | 0.004 (0.007) |
| Percentage tree cover | 0.006 (0.006) | 0.009 (0.006) | 0.019 (0.018) |
| Population density | 0.003 (0.005) | 0.006 (0.006) | 0.014 (0.014) |

Table S2. Comparison of social media SDM outputs generated with data of varying levels of spatial precision. Values represent the Spearman’s correlation coefficient between habitat suitability values from models built with a spatial precision threshold of 1, 2, 5, 10km^2^ and any data.

|  | All | 10km^2^ | 5km^2^ | 2km^2^ | 1km^2^ |
| --- | --- | --- | --- | --- | --- |
| All | 1 |  |  |  |  |
| 10km^2^ | 0.991 | 1 |  |  |  |
| 5km^2^ | 0.979 | 0.985 | 1 |  |  |
| 2km^2^ | 0.960 | 0.970 | 0.980 | 1 |  |
| 1km^2^ | 0.968 | 0.974 | 0.973 | 0.976 | 1 |

a) Traditional


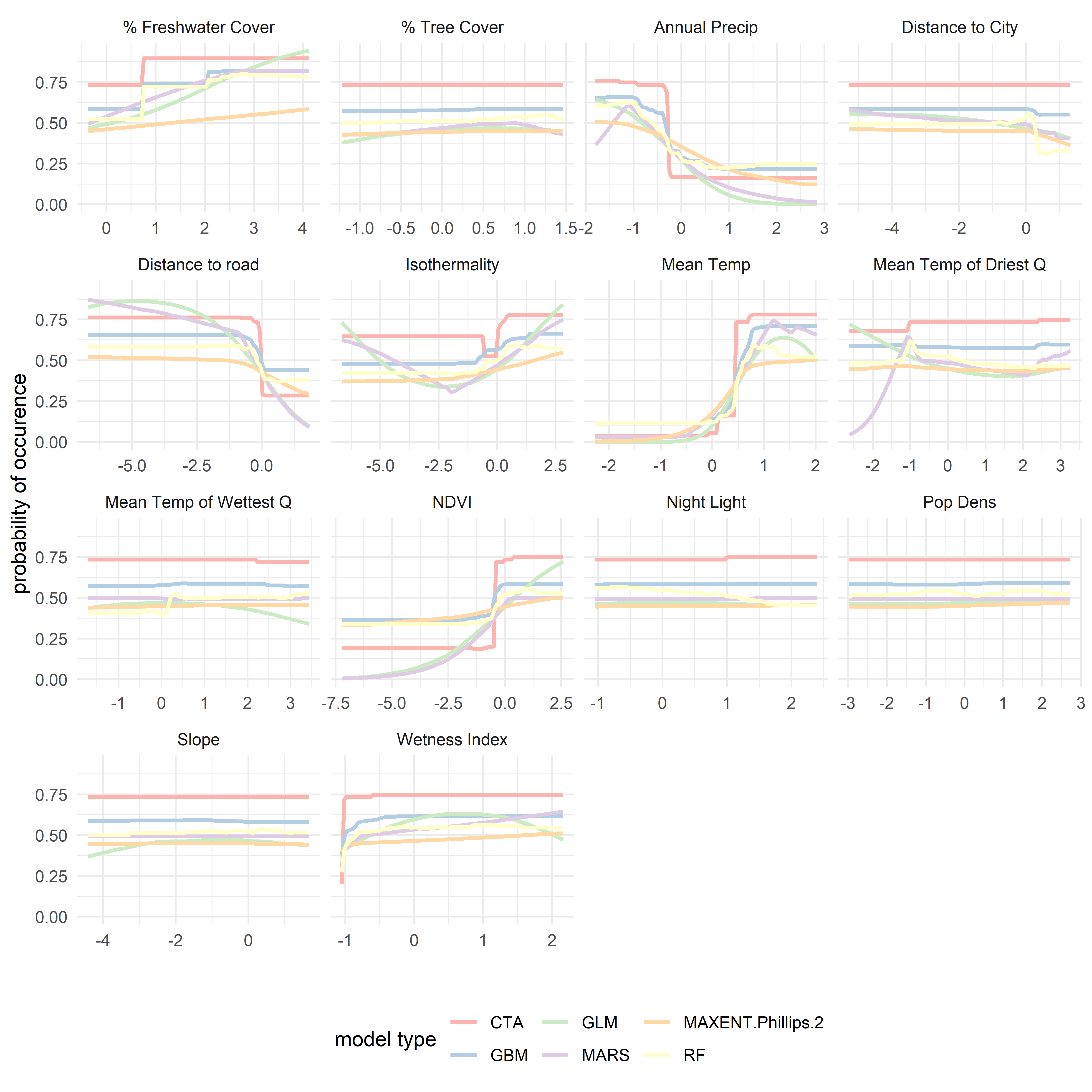


b) Citizen Science


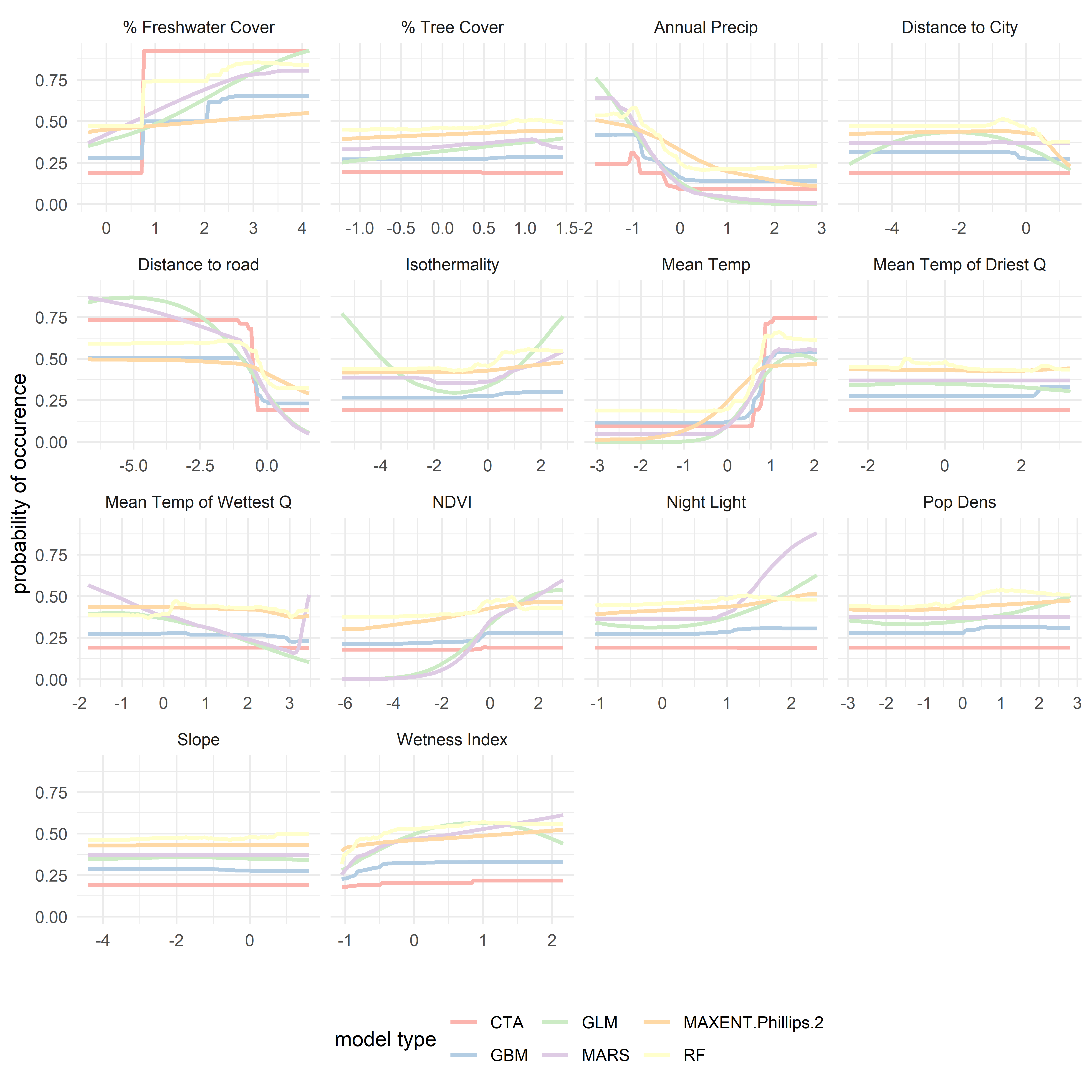


c) Social Media


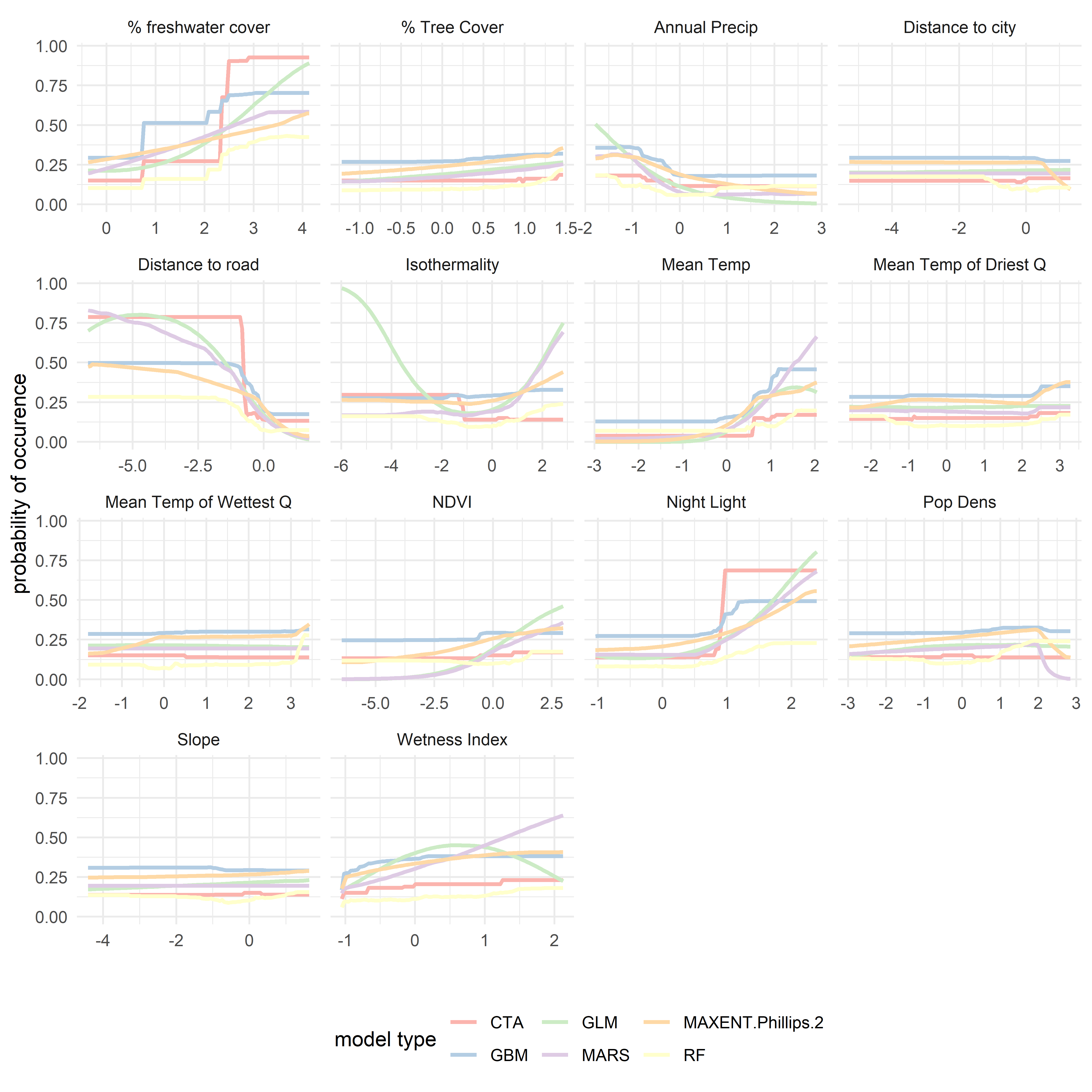


Fig. S1. Summary of response curves from biomod2 species distribution model outputs for a) traditional, b) citizen science, and c) social media occurrence data. Lines show median response curves for each modelling algorithm, filtered to only include models that were kept in the final ensemble model. All variables are scaled to a mean of 0 and a standard deviation of 1. See Table S1 for full variable names.


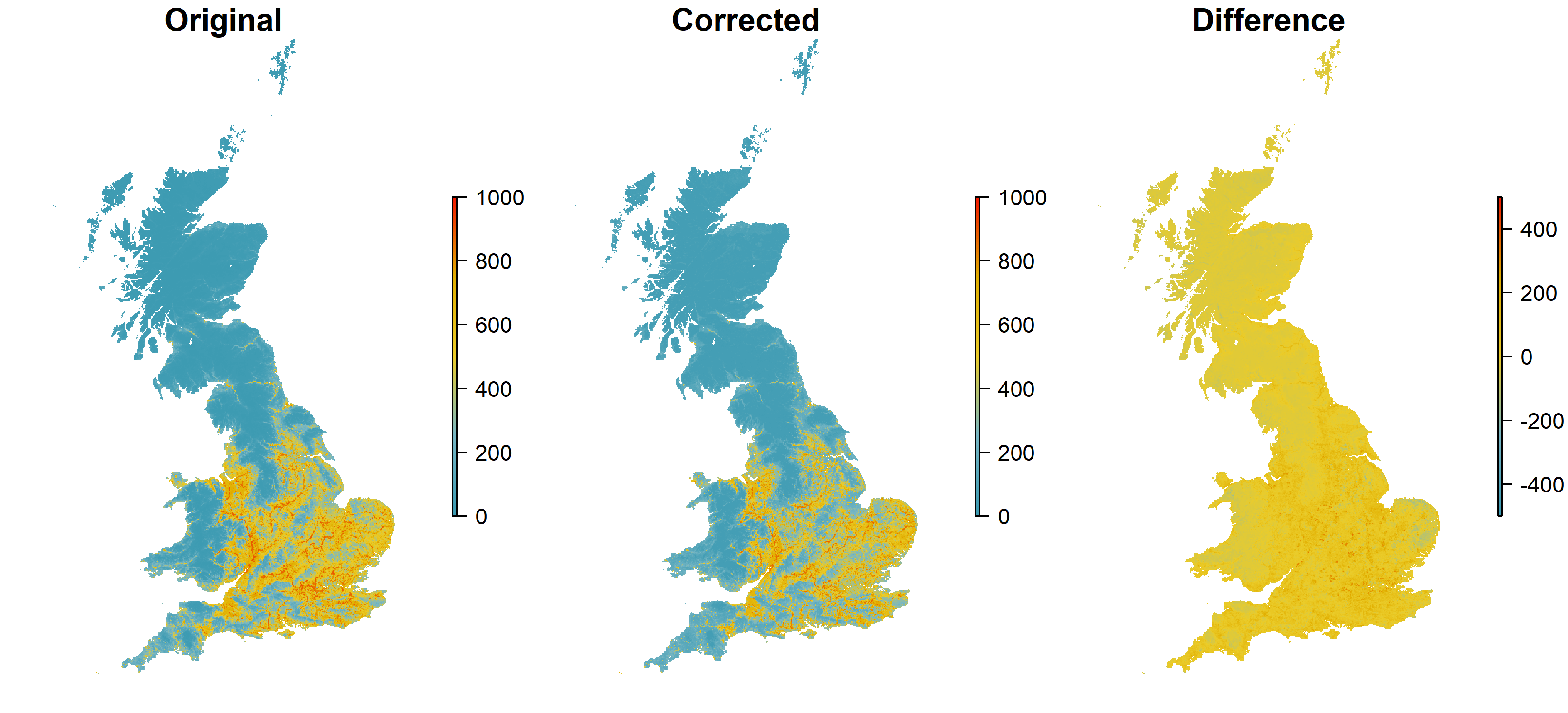


**Traditional**


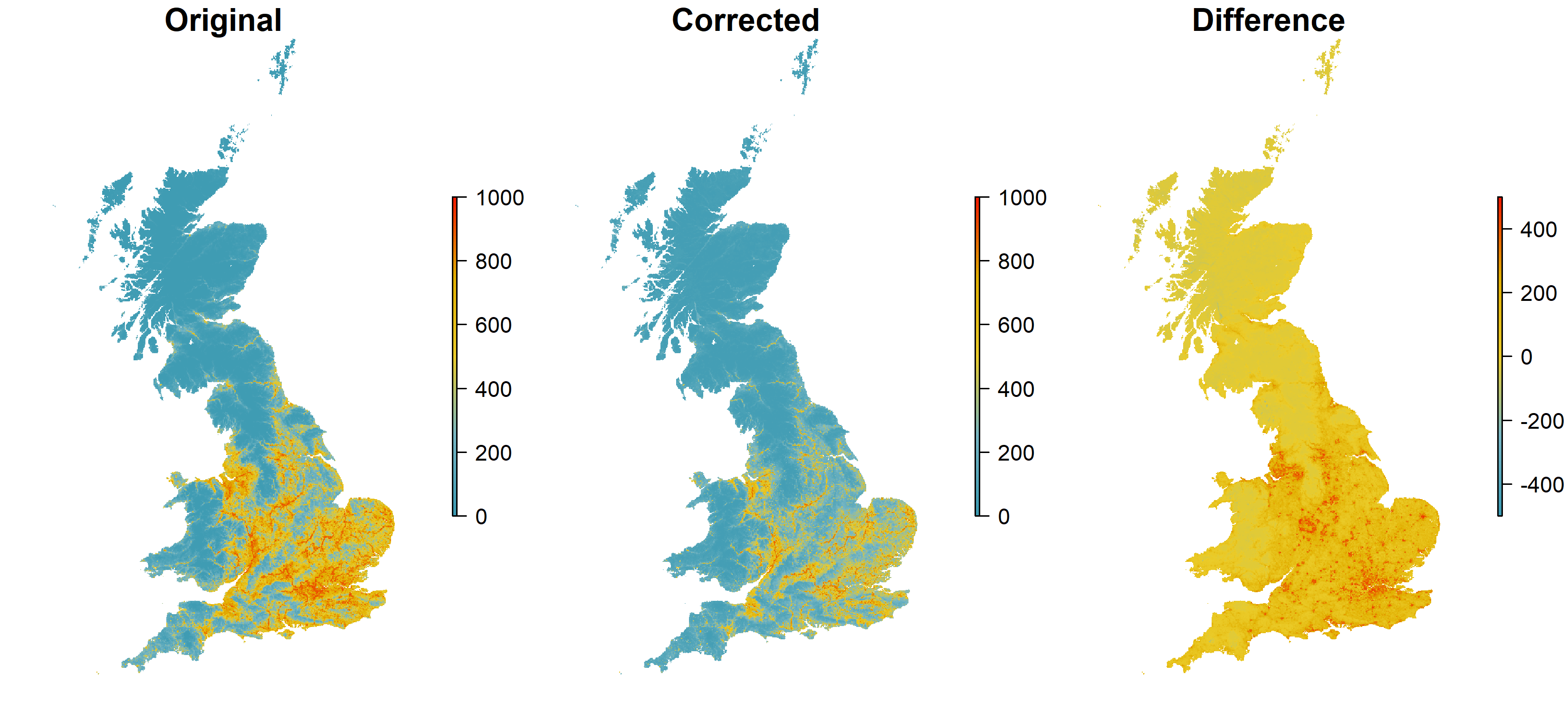


**Citizen Science**


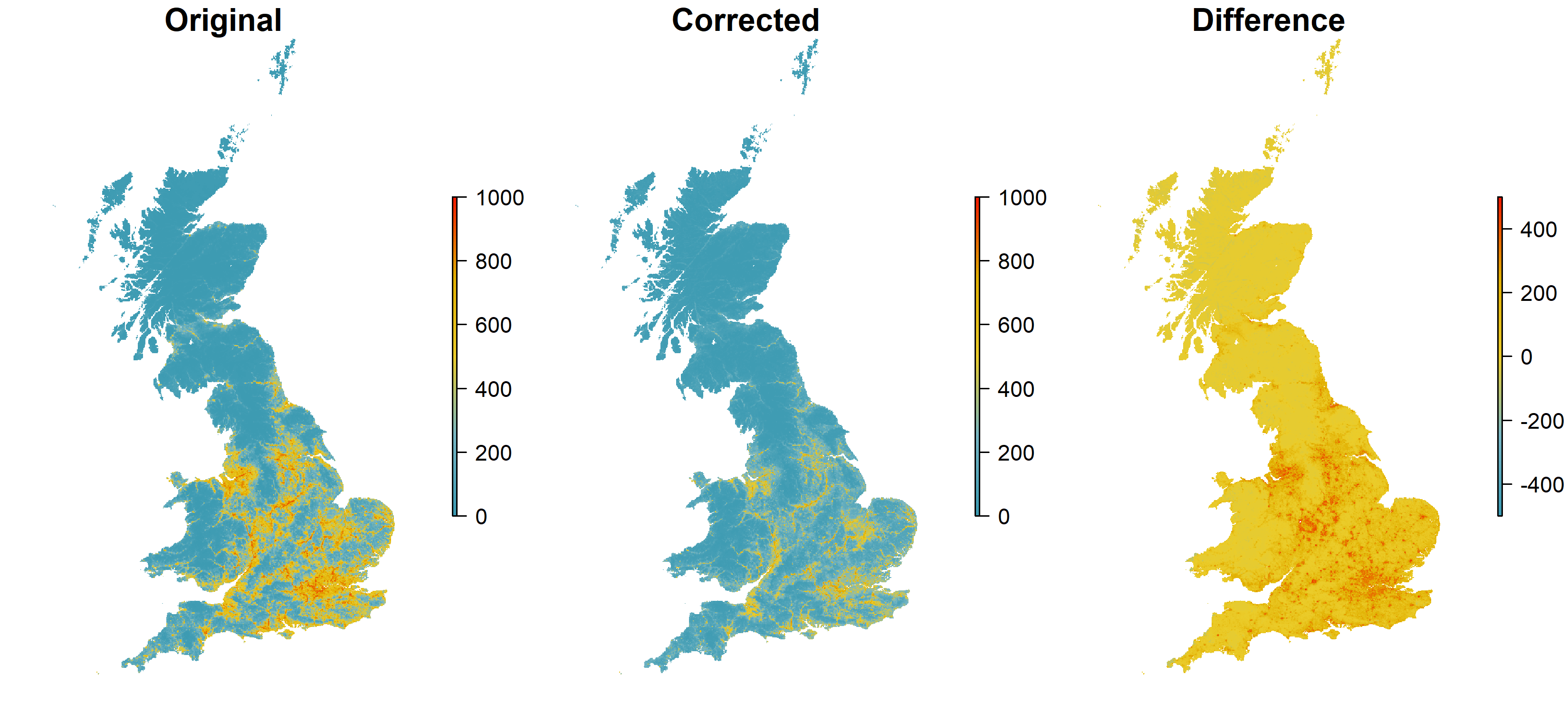


**Social Media**

Fig. S2. Comparison of SDM outputs generated with and without corrections for sampling bias for a) traditional b) citizen science and c) social media models. Left hand figures show the uncorrected model, built with environmental variables but not with sampling bias variables, centre figures show the corrected model, built with both environmental and sampling bias variables. Right hand figures show the difference between corrected and uncorrected models, positive values indicate that habitat suitability was higher in the uncorrected model.


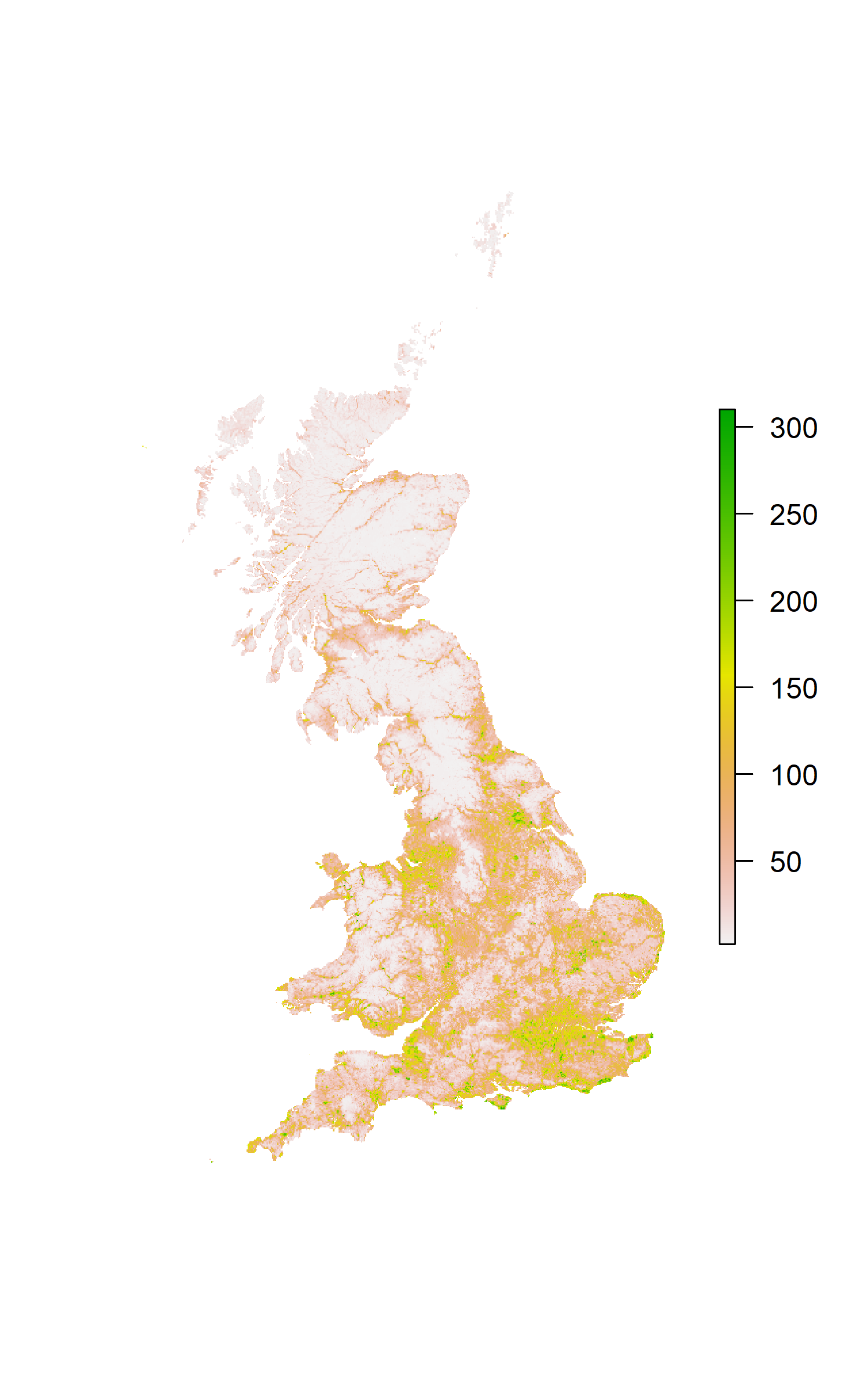


Fig. S3. Comparison of social media SDM outputs generated with data of varying levels of spatial precision. Values represent the range in habitat suitability values from models built with a spatial precision threshold of 1, 2, 5, 10km^2^ and any data. A score of 0 indicates that all models had high agreement, positive scores indicate high disagreement.
